# Expression of Concern: Priorities for intervention of childhood stunting in northeastern Ethiopia: A matched case-control study

**DOI:** 10.1371/journal.pone.0329790

**Published:** 2025-08-06

**Authors:** The PLOS One Editors

This article [1] was identified as one of a series of articles for which the *PLOS One* Editors have concerns about authorship and adherence to the journal’s first and second publication criteria.

In addition, contrary to the declaration in the Data Availability statement, the original raw data files supporting the article’s results were not provided with the article, and the authors have not provided these data following editorial request. As the original raw data files supporting the article’s results have not been made available, this article [1] does not comply with the PLOS Data Availability policy.

We regret that the issues were not addressed prior to the article’s publication.
