# Supplementary material for: Priorities for intervention of childhood stunting in northeastern Ethiopia: A matched case-control study
Source: PLoS One. 2020 Sep 24;15(9):e0239255. doi: 10.1371/journal.pone.0239255 (PMC7514084; doi:10.1371/journal.pone.0239255)
Supplement: S1 File — (DOCX) [file pone.0239255.s001.docx]

| **Questionnaires ID.No……… Address……..... Location Woreda: Kebele: GPS location**   **Date of interview……………… Time started……… Time finished………. Code number………:** | | | |
| --- | --- | --- | --- |
| **s.no** | **Part I: Socio Economic and Demographic Factors** | |  |
| 101 | Age of the child in months | ___________ |  |
| 102 | Sex of the child | 1. Male 2. Female |  |
| 103 | Who gave cares for the child? | 1. Mother 2. Person other than mother |  |
| 104 | How many children of less than 5 years old were/ are found in your household? | _____________in number |  |
| 105 | Age of mother/care giver in years | _________years. |  |
| 106 | Marital status of mother | 1. Single 2. Married 3. Divorced 4. Window 5. Others (specify) _____________ |  |
| 107 | Ethnicity | 1. Amara 2. Tigray 3. Afar 4. Others (specify) ___________ |  |
| 108 | Religion | 1. Orthodox 2. Muslim 3. Others (specify) _________ |  |
| 109 | Educational status of mother | 1. Illiterate 2. Elementary 3. High school 4. Diploma 5. 1st degree & above |  |
| 110 | Educational status of father | 1. Illiterate 2. Elementary 3. High school 4. Diploma 5. 1^st^ degree & above |  |
| 111 | Occupational status of mother | 1. Civil servant 2. Own business 3. Wage work permanently 4. Wage work occasionally 5. Trade 6. Household work 7. Other (specify) ________ |  |
| 112 | Family income per month in Birr | ___________ ETB |  |
| 113 | Total family members | ___________in number |  |
| 114 | Who has got food priority in your household? | 1. Father 2. Mother 3. Children 4. All equal 5. Others (specify) ______________ |  |
|  | **Part II: Health Care Related Factors** |  |  |
| 201 | How many total births did you give during your life? | ___________in number |  |
| 202 | What was the time gap between the last two pregnancies (if the mother has two and above children)? | ___________in number |  |
| 203 | What was the type of the pregnancy for this baby? | 1. Unplanned and Unwanted 2. Unplanned but wanted 3. Planned and Wanted |  |
| 204 | Did you have antenatal care follow up during pregnancy of this baby? | 1. Yes 2. No |  |
| 205 | If yes to question number 204, how many times did you visit the health institution for antenatal care? | 1. 1 to 2 times 2. 3 to 4 times 3. More than 4 times 4. I don’t remember |  |
| 206 | Where did you give birth to this baby? | 1. At home 2. Health institution |  |
| 207 | What was the birth weight of the baby? | 1. <2.5 kg 2. 2.5-4kg 3. >4kg |  |
| 208 | Did you have to post natal care follow up? | 1. Yes 2. No |  |
| 209 | Does the baby completed (on the right track of) immunization? | 1. Yes 2. No |  |
| 210 | What was/were the repeated typing of child illness two weeks before? (Possible to select more than one) | 1. Not ill 2. Respiratory infections 3. Diarrhea 4. Fever 5. Others (specify) _________ |  |
|  | **Part III: Child Feeding Practices Factors** |  |  |
| 301 | Have you ever breast feed the child since birth? | 1. Yes 2. No | If no, go to Q_306_ |
| 302 | How long after birth did you first put the baby to the breast feeding? | 1. <=1 hour 2. 1-3 hours 3. >3 hours 4. I don’t remember |  |
| 303 | Did you feed the baby colostrum? | 1. Yes 2. No |  |
| 304 | Did you give anything to drink after birth before feeding breast milk? | 1. Yes 2. No |  |
| 305 | For how long was your baby on exclusive breastfeeding? | ___________months |  |
| 306 | How much breast feedings per day was given to baby while he/she was in exclusive breastfeeding? | ____________times |  |
| 307 | Did you give your baby any feedings besides breastfeeding? | 1. Yes 2. No |  |
| 308 | If yes, for Q_309_ at what age was started? | __________months |  |
| 309 | What was the method of complementary feeding? | 1. Bottle  2. Hand  3. Spoon  4. Other (specify) ______ |  |
| 310 | How many times did/ do you give complementary foods/liquid per 24 hours? | _______times per 24 hours |  |
| **Part IV: Sanitation and Water Related Factors** | | | |
| 401 | What kind of toilet facility did/does your household use? | 1. Has no toilet 2. Pit latrine 3. VIP latrine 4. Water Flush 5. Others (specify) |  |
| 402 | When did you wash your hand, usually? (Possible to select more than one) | 1. After using the toilet 2. Before food preparation 3. After cleaning, child 4. After meals 5. I don’t remember 6. Other (specify) _____________ |  |
| 403 | What did you use to wash your hand? | 1. Only water 2. Sometimes with soap 3. Always with soap 4. Others (specify) _________ |  |
| 404 | What was/is the main source of drinking water for the family members of the household? | 1. Tap/public 2. Borehole 3. Protected well/spring 4. Unprotected source |  |
| 405 | How did your household primarily dispose household waste? | 1. Collected by municipality 2. Buried 3. Dumped in street/open space 4. Burned 5. Others (specify) ________ |  |
