# Supplementary material for: Priorities for intervention of childhood stunting in northeastern Ethiopia: A matched case-control study
Source: PLoS One. 2020 Sep 24;15(9):e0239255. doi: 10.1371/journal.pone.0239255 (PMC7514084; doi:10.1371/journal.pone.0239255)
Supplement: S2 File — (DOCX) [file pone.0239255.s002.docx]

## Amharic (local language translated) Questionnaire

| የመጠይቁ መ.ቁ…… አድራሻ ……... የጤና ተቋሙ ስም ............ ቃለ መጠይቅ የተደረገበት ቀን ……………… የተጀመረበት ሰዓት……… ያለቀበት ሰዓት………. ኮድ ………. | | | | | |
| --- | --- | --- | --- | --- | --- |
| ተ.ቁ | | **ቃለ መጠይቅ** | | መለያ ቁጥር | ዝለል |
| **ክፍል አንድ : ሥነ-ሕዝብና ኢኮኖሚን የተመለከተ ጥያቄ** | | | | |  |
| 101 | | የህፃኑ/ኗ ዕድሜ ……………… |  | |  |
| 102 | | የህፃኑ/ኗ ፆታ | 1. ወንድ 2. ሴት | |  |
| 103 | | ህፃኑን የሚንከባከበው ማነው? | 1. እናት 2. ሌላ (…………) | |  |
| 104 | | በቤተሰብ ውስጥ ዕድሜያቸው ከ 5 ዓመት በታች የሆኑ ህፃናት ስንት አሉ?…… | | | |
| 105 | | የእናት አድሜ: ……………… | | |  |
| 106 | | ሃይማኖትሽ ምንድን ነው? | 1. ኦርቶዶክስ 2. ሙስሊም 3. ካቶሊክ 4. ፕሮቴስታንት 5. ሌላ: ……………… | |  |
| 107 | | የቤተሰብሽ ብዛት ስንት ነው?……………… | | |  |
| 108 | | ብሔርሽ ምንድን ነው? | - 1. አማራ   2. ትግሬ   3. ኦሮሞ   4. ሌላ:………… | |  |
| 109 | | የጋብቻ ሁኔታ | 1. ያላገባች 2. ያገባች 3. ባሏ የሞተባት 4. የተፋታች | |  |
| 110 | | የትምህርት ደረጃሽ ስንት ነው? | 1. ያልተማረች 2. 1^ኛ^ ደረጃ 3. 2^ኛ^ ደረጃ 4. ዲፕሎማ 5. 1^ኛ^ ዲግሪና በላይ | |  |
| 111 | | የባልሽ የትምህርት ደረጃ ስንት ነው? | 1. ያልተማረ 2. 1^ኛ^ ደረጃ 3. 2^ኛ^ ደረጃ 4. ዲፕሎማ 5. 1^ኛ^ ዲግሪና በላይ | |  |
| 112 | | ስራሽ ምንድን ነው? | 1. መንግስት ሰራተኛ 2. ገበሬ 3. የግል ስራ ያላት 4. ቋሚ የቀን ሰራተኛ 5. ጊዜያዊ የቀን ሰራተኛ 6. ነጋዴ 7. የቤት እመቤት 8. ጡረተኛ 9. ሌላ: ……………… | |  |
| 113 | | በቤተሰብ ውስጥ ምግብ ቅድሚያ ለማን ይሰጣል | 1. ለአባት 2. ለእናት 3. ለህፃን 4. ለሁሉም እኩል 5. ሌላ ………… | |  |
| 114 | | የቤተሰብሽ ወርሃዊ ገቢ ስንት ነው?: …………… ብር | | |  |
| **ክፍል ሁለት: የግልና የውሃ ንፅህና** | | | | | |
| 201 | | በእዴሽ ስንት ወልደሻል | ……………… | |  |
| 202 | | በስንት ቀን አራርቀሽ ትወልጃለሽ? (ከ 1 ህፃን በላይ ላላት እናት) | ___________ | |  |
| 203 | | የእርግዝናው ዓይነት? | 1. ያልታቀደ የማይፈለግ 2. ያልታቀድ የሚፈለድ 3. የታቀደ የሚፈለግ | |  |
| 204 | | የቅድመ ወሊድ ክትትል አድርገሻል? | 1. አዎ 2. የለም | |  |
| 205 | | መልስሽ አዎ ከሆነ፣ስንት ጊዜ ክትትል አደረግሽ | 1. 1-2 ጊዜ 2. 3-4 ጊዜ 3. ከ 4 በላይ | |  |
| 206 | | ልጅሽን የት ወለድሽ | 1. ጤና ተቋም 2. ቤት | |  |
| 207 | | ሲወለድ ልጅሽ ስንት ኪሎ ነበር | 1. <2.5 ኪ.ግ 2. 2.5-4ኪ.ግ 3. >4ኪ.ግ | |  |
| 208 | | የድህረ ወሊድ አገልግሎ አድርገሻል | 1. አዎ 2. የለም | |  |
| 209 | | ህጻኑ ክትባት በስነስርዓት እየወሰደ ነው | 1. አዎ 2. የለም | |  |
| 209 | | ልጅሽ በተደጋጋሚ በየትኛው በሽታ ይታመማል | 1. አሞት አያውቅም 2. የመተንፈሻ አካሉን 3. ተቅማጥ 4. ትኩሳት 5. ሉላ | |  |
| **ክፍል ሶስት: የህፃኑ አመጋገብን የተመለከቱ ጥያቄዎች** | | | | | |
| 301 | | ጡት አትብተሸው ታውቂያለሽ? | 1. አዎ 2. የለም | |  |
| 302 | | በወለድሽ በስንት ሰዓት ጡት አስጀመርሽው? | 1. በ1 ሰዓት ውስጥ 2. 1-3 ሰዓት 3. ከ 3 ሰዓት በላይ | |  |
| 303 | | ልጅሽ እንገር ጠብቷል? | 1. አዎ 2. የለም | | የለም ከሆነ ወደጥ_306 ሂድ_ |
| 304 | | ከጡት በፊት ልጅሽ ምግብ ቀምሷል? | 1. አዎ 2. የለም | |  |
| 305 | | ልጅሽ ጡት ብቻ ለስንት ወር ጠባ? ………………… | | |  |
| 306 | | በቀን ስንት ጊዜ ይጠባ ነበር? ………………… | | |  |
| 307 | | ከጡት በተጨማሪ ምግብ ሰጥተሸዋል? | - 1. አዎ   2. የለም | |  |
| 308 | | መልስሽ አዎ ከሆነ፣ በስንት ወር አስጀመርሽው? ……………… | | |  |
| 309 | | ተጨማሪ ምግብ በቀን ስንቴ ይወስድ ነበር? ……………… | | |  |
| 310 | | በምን ይመገብ ነበር? | 1. በጡጦ 2. በእጅ 3. በማንኪያ 4. ሌላ፡………………… | |  |
| **ክፍል አራት: ንጽህናን የተመለከቱ ጥያቄዎች** | | | | |  |
| 401 | መጸዳጃ ቤት አላችሁ? | | 1. አዎ 2. የልም | |  |
| 402 | ካለ ምን ዓንነት ነው?…………… | |  | |  |
| 403 | አዎ ከሆነ ስንቴ ገጥሞሽ ያውቃል? …………… | | | |  |
| 404 | እጅሽን መቼ ትታጠቢያለሽ? (ከአንድ በላይ መልስ ይቻላል) | | 1. ከመጸዳጃ መልስ 2. ምግብ ለማዘጋጀት በፊት 3. ህፃኑን ካፀዳዳሁ በኋላ 4. ከምግብ በኋላ 5. አልታጠብም | |  |
| 405 | እጅሽን በምን ትታጠቢያለሽ? | | 1. በዉህ ብቻ 2. ሁሌ በውሃና በሳሙና 3. አልፎ አልፎ በውሃና በሳሙና 4. ሌላ …………… | |  |
| 406 | ለመጠጥ ውሃ የምትጠቀሙት ከየት ነው? | | 1. ከቧንቧ 2. ካልተጠበቀ ጉድጓድ 3. ከተጠበቀ ጉድጓድ | | አዎ ከሆነ ወደጥ_412 ሂድ_ |
| 407 | ደረቅ ቆሻሻ የምታስወግዱት እንዴት ነው? | | 1. በማዘጋጃ ቤት በኩል 2. በመቅበር 3. ሜዳ ላይ መጣል 4. ማቃጠል 5. ሌላ ………… | |  |

**ክፍል ሦሥት፡ ህፃኑ የተመገበው/ችውን የተለያዩ የምግብ ዓይነት መጠየቂያና መመዝገቢያ ቅጽ**

እባክሽ ትላንትና ከጠዋት ጀምሮ ልጅሽ የበላውን/ችውንና የጠጣውስ/ችውን የምግብ ዓይነት ከመጀመሪያው ጀምረሽ በዝርዝር ንገሪኝ፡፡ የተጠቀሱትን ምግብና መጠጦች በሚከተለው ሰንጠረዥ ይሙሉት፡፡ የጋራ ምግብ ከሆነ ከምን ከምን እንደተዘጋጀ በመጠየቅ ይሙሉት፡፡ ተጠያቂዋ ስትጨርሰ ትላንትና ከቤት ውጪ የመገበችው/ቻት ወይም ያጠጣችው/ቻት የምግብ ዓይነት ካለ በማስታወስ እንድትነግርህ አደርግ/ጊ፡፡

**ሰንጠረዥ 4፡ ትላንትና በ 24 ሰዓት ውስጥ የተመገበችውን ምግብ መመዝገቢያ ቅፅ**

| **ቁርስ** | **ሻይ ሰዓት** | **ምሳ** | **መክሰስ** | **እራት** | **ከራት በኋላ** |
| --- | --- | --- | --- | --- | --- |
|  |  |  |  |  |  |

**ሰንጠረዥ 6፡ የምግብ ድግግሞሽ ጊዜ መመዝገቢያ ቅፅ፡፡**

| **የምግብ ዝርዝር** | **መመገቢያ ጊዜ** | | | | |
| --- | --- | --- | --- | --- | --- |
|  | በየቀኑ | በየሁለት ቀኑ | በሳምንት ከ 1-2 ጊዜ | በሁለት ሳምንት አንዴ | ምንም አልወስድም |
| ቀይ ሥጋ |  |  |  |  |  |
| ቅጠላቸው አረንጓዴ የሆኑ አትክልት (ጎመን፣ሰላጣ፣ ቆስጣ ወዘተ.) |  |  |  |  |  |
| ፍራፍሬ (ማንጎ፣ ፓፓያ፣ሙዝ፣ወዘተ) |  |  |  |  |  |
| ጥራጥሬ( ባቄላ፣ አተር፣ ምስር፣አኩሪ አተር ወዘተ) |  |  |  |  |  |
| የጉበት ሥጋ |  |  |  |  |  |
| እንቁላል |  |  |  |  |  |
| ከጤፍ የተዘጋጀ ምግብ |  |  |  |  |  |
| ወተትና የወተት ተዋጽዖ |  |  |  |  |  |

ቃለ መጠይቅ ያደረገው ሰው ስም ………………..…….. ፊርማ ………ቀን ………………
