# Supplementary material for: Priorities for intervention of childhood stunting in northeastern Ethiopia: A matched case-control study
Source: PLoS One. 2020 Sep 24;15(9):e0239255. doi: 10.1371/journal.pone.0239255 (PMC7514084; doi:10.1371/journal.pone.0239255)
Supplement: Attachment — Submitted filename: Round 2-Rebuttal letter (Response to reviewers.doc [file pone.0239255.s003.doc]

Date 26 December, 2019

**Manuscript ID:** PONE-**-D-17-43531**

**Manuscript title:** Determinants of stunting among children aged 6-59 months in Kemissie City Administration, North Eastern Ethiopia: A community-based matched case-control study.

Authors: **Tadesse *et al.***

Dear editor,

We were pleased to know that our manuscript was rated as potentially acceptable for publication in PLoS ONE, subject to adequate revision as requested by the reviewers, editors and journal in-house editors. Based on the instructions provided in your letter, we uploaded the file of the rebuttal letter; the marked up copy of the revised manuscript highlighting the changes made in the original submitted version and the clean copy of the revised manuscript.

We have revised the manuscript by modifying/editing the abstract, introduction, methods, results, discussion and other sections, based on the comments made by the reviewers and using the journal guidelines. Accordingly, we have marked in red color all the changes made during the revision process. Appended to this letter is our point-by-point response to the comments made by the reviewers.

We agreed with almost all the comments/questions raised by the reviewers and provided justification for disagreeing with some of them. We would like to take this opportunity to express our thanks to the reviewers for their valuable comments. We would like also to thank you for allowing us to resubmit a revision of the manuscript.

I hope that the revised manuscript is accepted for publication in PLoS ONE.

Sincerely yours,

Dr Metadel Adane

PhD in Water and Public Health in Addis Ababa University, Ethiopia

Lecturer and Researcher in Wollo University, Ethiopia
Phone: +251 (0) 945963080

E-mail address: [metadel.adane2@gmail.com](mailto:metadel.adane2@gmail.com)

**Answer for Academic editors and Reviewers**

First, we would like to thank all the reviewers for supplying us a relevant comment in order to make our manuscript upto the standared. We revised the masncuript accordingly.

Reviewer #2:

Question 1: Readers of this revised paper will still be unable to fully interpret it because the basic information about the extent of stunting of the cases and controls is not stated. There is no point in simply putting the data in a reply to reviewers if it is not also put in the paper. I suggest that it is added as a line to Table1: (Socio-demographic characteristics of children aged 6-59 months of age, Kemissie city administration, 2017), and in describing the children in the results under Socio demographic and economic characteristics, the HAZ score in terms of mean, SD and range of values should be included.

Answer:

In this study cases were defined as those children with height-for-age Z score of <-2SD. While controls were defined as children with height-for-age Z score of <-2SD. This definition was taken from WHO growth standards. Based on WHO internationally accepted growth standard, an HAZ score of >-1 could never be a case, rather it is a control. For further information you can review (*WHO* growth standards*, 2009*). We used the WHO growth standard (Z score <-2 SD or ≥ -2 SD). Based on your request, the mean HAZ score for the cases and controls was -2.79±.67 and -0.55±.92 respectively. Since the mean of SD is within your comment, it is possible to proceed with the analysis we performed. Therefore, as HAZ score goes to lowest value the severity of stunting is increasing and vice versa (see line 168-169).

Question 2: Note that Table 4 is mistakenly headed Table 1.

Thank you for the comments. We made the correction.

Question 3: The authors should seek advice on their use of English because the grammar requires improvement. Thank you for the suggestion of language improvement. We improved the language error and professional editors Dr Helmut Kloos and Lisa Penttila. We acknowledged the language editors at the acknowledgement section (See line 293).

Reviewer #3: The question on the PCA was not adequately addressed. The authors did not tell what variables were included in the PCA analysis. What were the summary components and how much variance was explained by the final components? Also what determines the wealth index of poor, medium and rich?

Answer:

Thank you for this key questions: PCA was conducted in order to calculate the wealth index by including assets of the community. We made correction by changing economic status to wealth index.After checking the assumptions of PCA, rural and urban household assest from Ethiopian demographic and health survey (EDHS, 2016) were used to conduct the variables which were included in the PCA analysis. This is variables include radio, television, electric mitad, frig, bed, roof made from corrugated iron sheet, wall made from cement, mobile, camel, ox, cow, goat, sheep, horse, baja, taxi, chair, table, sofa, lamp, shoes were the variables included in PCA analysis. The summary components were television, frig, mozbold bed, roof made from corrugated iron sheet, wall made from cement and 63.82% of variance was explained by the final components. By using the above five variables wealth index was computed by calculating the mean and finally the mean was divided in to three in order to categorize the study population as poor, medium and rich. In addition wealth index was not significant in the final model, that is why we did not include all the above issues in the main document **(Please see all this update 133-142)**.

**We would like to thank the reviewers and editors for evaluating our manuscript. We have tried to address all the concerns in a proper way and believe that our paper has improved considerably. We would be happy to make further corrections if necessary and look forward to hearing from you all soon.**
